# Supplementary material for: ProteinShader: illustrative rendering of macromolecules
Source: BMC Struct Biol. 2009 Mar 30;9:19. doi: 10.1186/1472-6807-9-19 (PMC2672931; doi:10.1186/1472-6807-9-19)
Supplement: Additional file 1 — ProteinShader program without source code. This compressed file contains the complete ProteinShader program including associated libraries, but no source code. A README.txt file gives an overview of the ProteinShader distribution, and the index.html file in the help subdirectory has directions on getting started with the program as well as a set of tutorials. [file 1472-6807-9-19-S1.zip › ProteinShader-beta-0_9_4-binary/help/api/org/proteinshader/structure/visitor/Visitable.html]

Visitable (ProteinShader API)


|  |  |  |
| --- | --- | --- |
| **Overview** \| **Package** \| **Class** \| **Use** \| **Tree** \| **Deprecated** \| **Index** \| **Help** | |  |
| **PREV CLASS**   **NEXT CLASS** | **FRAMES**    **NO FRAMES**     **All Classes** |  |
| SUMMARY: NESTED \| FIELD \| CONSTR \| METHOD | DETAIL: FIELD \| CONSTR \| METHOD |  |


---


## org.proteinshader.structure.visitor Interface Visitable

**All Known Implementing Classes:**: AminoAcid, Atom, BetaStrand, Bond, Chain, Drawable, Helix, Heterogen, Loop, Model, Region, Residue, Segment, Structure, Water

---

``` public interface Visitable ```

This interface requires an accept( Visitor ) method, which should be
used to call back to the Visitor so that the Visitor can perform one
or more operations on the Visitable object.

---

| **Method Summary** | |
| --- | --- |
| `void` | `accept(Visitor visitor)`             Accepts a Vistor so that a call back can be done. |

**Method Detail**

### accept

```
void accept(Visitor visitor)
            throws VisitorException
```

:   Accepts a Vistor so that a call back can be done.

    :   **Parameters:**: `visitor` - the Visitor to do a callback with. **Throws:**: `VisitorException` - if an error occurs while an object is being visited.


---


|  |  |  |
| --- | --- | --- |
| **Overview** \| **Package** \| **Class** \| **Use** \| **Tree** \| **Deprecated** \| **Index** \| **Help** | |  |
| **PREV CLASS**   **NEXT CLASS** | **FRAMES**    **NO FRAMES**     **All Classes** |  |
| SUMMARY: NESTED \| FIELD \| CONSTR \| METHOD | DETAIL: FIELD \| CONSTR \| METHOD |  |


---

# *Copyright © 2007-2008*
